# Advancing access to substance use prevention for foster youth through digital innovation: an open trial of fostrspace with court appointed special advocate programs

**DOI:** 10.1186/s12913-025-12811-9

**Published:** 2025-05-10

**Authors:** Marina Tolou-Shams, Johanna B. Folk, Margareth V. Del Cid, Jannet Lara Salas, Alison Czopp, Juan Carlos Gonzalez, Tylia Lundberg, Erin Michelle Turner Kerrison, Martha Shumway, Ann Wrixon, Robert Salgado, Jill Duerr Berrick

**Affiliations:** 1 Department of Psychiatry and Behavioral Sciences|Weill Institute for Neurosciences, University of California, San Francisco, Zuckerberg San Francisco General Hospital, Pride Hall 2540 23rd street, San Francisco, CA 94110 USA; 2 School of Social Welfare, University of California, Berkeley, 120 Haviland Hall, Berkeley, CA 94720 USA; 3 CASA of Contra Costa County, 2151 Salvio Street, Ste. 295, Concord, CA 94520 USA

**Keywords:** Digital behavioral health intervention, Foster youth, Substance use, Mental health

## Abstract

**Background:**

Adolescents in foster care report high rates of mental health needs, yet intervention access remains limited. Substance use commonly co-occurs with mental health symptoms, but availability of substance use services for foster youth is even more scant than mental health services. Technology has advanced access to behavioral health care across the lifespan, but only for certain sectors of the population. Little research focuses on leveraging technology to advance access for foster youth. We report open trial findings, as a precursor to launching a large-scale implementation science trial, on how a U.S. nationwide serving support system for foster youth, Court Appointed Special Advocates (CASA), might be leveraged to expand access to substance use prevention resources via the FostrSpace app. FostrSpace provides asynchronous resources and synchronous navigator, peer support, and direct clinical intervention. A concurrent 6-session ECHO® substance use prevention telementoring curriculum was co-developed as a FostrSpace implementation strategy with a 6-member CASA Advisory Board.

**Methods:**

Seven youth-CASA dyads enrolled in the open trial. We used a mixed-methods design (quantitative assessment and qualitative exit interviews) to assess feasibility and acceptability of ECHO® sessions (CASA-only) and the usability of the FostrSpace app (youth-only).

**Results:**

Six of seven of the youth accessed the app at least once, but a majority reported the app log-in process was burdensome and unappealing, thereby limiting them from frequently using the app. All youth rated the app features, design and content as appealing, helpful and relevant. ECHO® -FostrSpace session attendance was high (most attended 5 or more sessions) and CASAs found the content highly engaging and useful, especially regarding CASA-youth substance use communication skills.

**Conclusions:**

Technological barriers, such as log-in burden, can prevent youth in need from accessing relevant services and must be regularly assessed and resolved. Substance use education and skills-building for CASAs is novel and a viable implementation strategy to increase foster youth access to digital behavioral health services innovations. Substance use prevention content should be integrated within discussions on youth mental health and trauma to be most engaging and relevant. Findings are informing the subsequent hybrid implementation-effectiveness trial design of FostrSpace with 400 youth-CASA dyads across 10 CASA programs in California.

**Trial registration:**

Retrospectively registered.

**Supplementary Information:**

The online version contains supplementary material available at 10.1186/s12913-025-12811-9.

## Background

The more than 350,000 youth in foster care [1] across the United States are considered some of the most vulnerable, in part due to high behavioral health needs [2, 3]. Foster youth have high rates of untreated substance use (SU) and co-occurring mental health (MH) needs and recent studies have demonstrated the significant increase in general youth population behavioral health concerns during and following the COVID-19 pandemic [4, 5]. Population-wide studies among youth in the child welfare system, including foster youth, show high rates of untreated alcohol and illicit SU (47%), with nearly one-quarter meeting criteria for substance use disorder [6]. Youth in foster care are nearly five times more likely than youth never removed from their homes to misuse substances [7]. Substance use disorders occur more frequently in older foster youth [8], a subgroup with specific and often unmet needs as they transition to adulthood [9]. Social determinants of health (SDoH; e.g., child maltreatment, delinquency, interpersonal violence, family conflict, social disconnection) have been consistently identified as drivers of SU among foster youth [10], and may explain why those who use substances also experience high rates of co-occurring MH needs [7].

Despite connections to federal- and state-supported behavioral health services, foster youth are infrequently assessed for SU and/or linked to empirically supported treatment [11, 12]. Foster youth face multiple barriers to accessing SU services including stigma, lack of treatment options with support from peers with lived experience, and generic treatments that do not consider individuals’ challenges as unique and nuanced [13]. Over two-thirds (66%) of 11–14 year-old foster youth have clinically significant MH needs, yet only 26% utilize MH services [14]. Inequities in MH services access for this population are amplified among foster youth of color and LGBTQ + foster youth [15].

### Leveraging technology to increase access to care

Behavioral intervention technologies (BITs) hold great promise *as behavioral health support tools* to provide foster youth direct easy access to resources [16] that address upstream SDoH and co-occurring SU and MH needs. BITs are “websites, software, mobile application (app), and sensors designed to help users address or change behaviors, cognitions and emotional states” [17]. BITs focused on youth SU prevention and intervention are acceptable and feasible to youth [18], increase access to care for foster youth [13] and other underserved populations (e.g., youth in the juvenile legal system) [19, 20], and reduce SU and MH symptoms [13, 16]. 

From 2021 to 2022, our research team used a participatory informatics approach [21] to co-design a BIT for foster youth. FostrSpace is a behavioral health and wellness app developed for and by individuals with lived experience in the foster care system to reduce stigma associated with accessing MH and co-occurring SU related care and resources (Folk JB, Gonzalez JC, Del Cid MV, McBride E, Lundberg T, Czopp A, et al: Co-designing a digital health app for foster youth: lessons learned from FostrSpace, under review). *Participatory informatics* combines best practices from Community Partnered Participatory Research (e.g., equity, power sharing) and user-centered design (e.g., active user participation in design) to create apps, including to promote community resilience [21] and for diverse community MH resource needs related to the COVID-19 pandemic [22]. Users are included as equitable members throughout the design process.

The FostrSpace co-design group (the FostrSpace Advisory Board, or FAB) embraced a *SDoH framework* for designing all aspects of the app and were guided by the World Health Organization definition of SDoH as “the conditions, in which people are born, grow, work, live and age and the wider set of forces and systems shaping the conditions of daily life [23]” SDoH, which overlap with social determinants of mental health, include domains such as economic stability, education access and quality, healthcare access and quality, neighborhood and built environment, and social and community context. Research shows tremendous impact of these factors on health outcomes [24], including negative impact on the ability of youth exiting foster care to successfully navigate systems to address health needs [25]. Consistent with participatory co-design, the FAB determined the most relevant forces and systems shaping the conditions of their daily life as foster youth, and in particular, impacting what they termed as “emotional wellness” [26]. The SDoH framework was used to inform decisions about what to include on a SDoH checklist of needs curated resources that should be available to foster youth users, and other areas of foci in the app. The resultant FostrSpace app is a “one stop shop” providing access [27] to a curated resource directory, psychoeducational materials, peer support, and a behavioral health care team (i.e., navigator, licensed clinician) who can support with resource linkage or provide direct SU and MH intervention via telehealth.

### Implementing FostrSpace within Court Appointed Special Advocates programs

Court Appointed Special Advocate (CASA) programs advocating for the needs of foster youth are well-established and widespread, but lack standardized training in empirically supported SU prevention practices. CASA was developed in 1977 and over 900 programs around the United States serve more than 240,000 youth annually [28]. CASAs are adult volunteers who work directly with youth in an ongoing, typically one-to-one, weekly, in-person, and long-term (average 18 months) capacity to advocate for appropriate resources (e.g., therapeutic, educational, housing) while youth are involved in the child welfare system. Despite regular contact, CASAs have limited ability to address behavioral health needs or provide specific behavioral health services, given their backgrounds as non-providers (i.e., most have no prior behavioral health training) and the scope of CASA programs.

CASA programs are a critical window of opportunity to reach foster youth for SU prevention services, and scholars have identified the need to conduct more CASA program research to better understand the ways in which CASAs can be leveraged to improve foster youth outcomes [29]. Data suggest gatekeeper training programs are necessary for non-MH professionals to successfully identify need for and make referrals to appropriate behavioral health services; this has been predominantly studied in suicide prevention services [30], but a review of such programs for youth MH services more generally concludes with scientific need to assess help-seeking through service utilization data, behavioral uptake (e.g., skills for making appropriate referrals), and symptom and well-being measurements [31]. The Extension for Community Healthcare Outcomes (ECHO®) is a telementoring, learning collaborative (peer and expert consultation) model which has been used for gatekeeper training programs in suicide prevention [30], SU [32], and co-occurring disorders [33], and is one promising model for supporting CASA volunteers in learning about SU prevention. The ECHO® model was established in 2003 and is now disseminated and implemented worldwide with over 900 ECHOs® around the world in areas including specialty medicine (e.g., infectious disease), pediatric primary care, community-based behavioral health, schools, and prison education [34]. 

### Current study

This paper reports findings from a 12-month trial development (adaptation and open trial) phase, conducted as a precursor to a Hybrid II implementation-effectiveness trial. The Hybrid II trial will test the effectiveness of ECHO® as an implementation strategy within CASA programs to increase *reach* (use) of the FostrSpace app with CASA youth, ages 13–20 years and *effectiveness* of the FostrSpace app in preventing youth SU across 10 CASA programs with 400 CASA-youth dyads (*N* = 800 participants total). The aims of the open trial were to: (1) make any necessary adaptations for adolescent substance use prevention in the FostrSpace app, as informed by participating foster youth and CASA volunteers; (2) co-design the implementation strategy (ECHO telementoring sessions) content and approach with CASA volunteers; and (3) conduct an open trial (run-through) of the FostrSpace-CASA design with minimum of 5 youth-CASA dyads in preparation for the full trial. Open trial data (quantitative assessment and qualitative exit interview) were used to identify whether milestones were met (e.g., 5 CASAs attended at least 75% of ECHO-FostrSpace sessions, 4 of 5 CASAs rated high satisfaction with ECHO sessions, 5 youth rated high acceptability and satisfaction with the FostrSpace app), and to inform any further final adaptations to the FostrSpace app, the ECHO implementation strategy and/or overall study design prior to launching the full trial.

## Methods

### Overview and timeline

In partnership with one CASA program, through a participatory co-design and mixed-methods approach including individual interviews, collaborative meetings with the FAB, a CASA Advisory Board (CASAAB), and direct observation, we spent 3 months adapting the FostrSpace app to expand SU prevention content and develop the ECHO®-FostrSpace (ECHO-FS) curriculum. An existing ECHO SU and co-occurring trauma curriculum successfully implemented with child and adolescent behavioral health providers serving systems-impacted youth served as a starting point for the ECHO-FS curriculum development [35]. We conducted a 3-month open trial with 14 participants (7 youth, 7 CASAs). The open trial intervention tested included a 3-month open trial of access to the FostrSpace app for CASA youth participants and the CASA program implementation strategy: a FostrSpace 101 Informational Session (30 min) plus 6, 90-minute ECHO-FS telementoring sessions delivered every other week for 3 months to CASAs. All study procedures were approved by the Institutional Review Board at [masked for submission].

### Advisory boards

For the open trial, the FAB was expanded from 4 to 6 members. The FAB met with the study team for six co-design meetings to identify areas of adaptation for SU prevention content that was then relayed to the technology developers. FAB members were all hired as UCSF staff and paid their hourly rate for meeting participation. The CASAAB was newly formed with 6 members who had been serving as CASA volunteers from 1 to 20 years and from a range of professional backgrounds (e.g., attorney, writer, school staff, retired college counselor). The CASAAB met with the study team for 6, 120-minute meetings over a 3-month period to develop the SU prevention content and approach for the ECHO-FS curriculum. CASAAB members were paid $100 per meeting.

### Open trial participants

*CASAs* were eligible if they worked with a youth between the ages of 13–20 years who was interested in participating with them. Exclusion criteria include being non-English speaking and if their youth was within 3 months of leaving the CASA program. *Youth* were between the ages of 13–20 years with an assigned CASA volunteer who was interested in participating with them. Exclusion criteria include being non-English speaking, within 3 months of leaving the CASA program and/or cognitive impairment that precludes ability to provide informed consent.

### Procedures

#### Recruitment

 CASA leadership distributed a flyer describing the open trial study opportunity to all CASA volunteers and interested CASAs were approached by study staff to determine eligibility and obtain informed consent. Study staff followed up with youth separately about interest in participation after CASAs provided contact information for their youth; both CASA and youth had to agree to participate for the dyad to be enrolled. CASA and youth completed independent informed consent processes (independent minor consent is allowable for 12 years and older in the state of CA, i.e., additional parent/legal guardian consent was waived). 

#### Quantitative assessments

Youth and CASA open trial participants completed quantitative assessments (lasting up to 45 min) using REDCap electronic data capture tools, a secure, web-based software platform [36, 37]. Assessments provided descriptive information about open trial participants and served as a pilot of the assessments to be used in the upcoming implementation-effectiveness trial. Youth and CASAs were each compensated $30 for completing the quantitative assessment. 

Youth completed a one-time assessment at enrollment which included: *primary and secondary outcome measures of SU* (Texas Christian University or TCU Drug Screen 5 [38] with opioid use supplement, cigarette smoking and vaping); *SU attitudes*,* beliefs*,* willingness and intentions* (e.g., Alcohol Expectancy Questionnaire [39]); *psychiatric symptoms* (Brief Symptom Inventory [40], National Stressful Events Survey PTSD Short Scale [41]); and hypothesized youth-related moderators, such as background characteristics, SDoH (education, housing, employment), youth/CASA relationship quality, trauma, ethnic identity, stigma, and peer SU. Throughout the open trial, data were also collected from the FostrSpace app on hypothesized mechanisms of change, including app utilization by youth (e.g., number of app log-in’s, days of use, time spent using app, number and type of features used e.g., chat use (peer/FAB), chat use (navigator), and number of clicks on SU prevention content (e.g., SU resources requested). Youth were also asked to complete brief user feedback surveys (feasibility/acceptability/satisfaction) after 30 and 60 days of open trial experience with the FostrSpace app [using the Systems Usability Scale or SUS [42]] assessing FostrSpace app usability. 

CASA volunteers completed a one-time assessment at enrollment which included: *SU prevention related knowledge* (survey designed based on specific ECHO curriculum content and informed by Moore’s framework for evaluating educational activities [43]); *beliefs about youth SU* (adapted 9-item perceived risk of SU measure used in studies of similar risk perceptions, among parents, associated with youth SU [44–46]); *attitudes towards evidence-based practices* (adapted version of the Evidence-Based Practice Attitudes Scale-50 [47]); *self-efficacy using empirically-supported practices* (adapted 19-item self-efficacy scale by Arora et al. [48]); *compassion satisfaction and fatigue* (Professional Quality of Life [49]); and hypothesized CASA-related moderators such as background characteristics. CASA volunteers were also asked to complete brief feedback surveys (feasibility/acceptability/satisfaction) after 30 and 60 days of open trial experience with the ECHO-FS. 

#### Qualitative exit interviews

Youth and CASAs were invited to separately complete brief 30–60 min exit interviews about their open trial experience. Interview guides were developed by the study team specifically for open trial evaluation purposes (see Supplemental files).

### Finalized intervention (FostrSpace app) and implementation strategy (ECHO-FostrSpace)

Table 1 presents details of FostrSpace features and content co-designed with the FAB, including adaptations (in italics) made to include more SU prevention content (e.g., in Resource Directory and Missions).

**Table 1:** FostrSpace app features and content

Type	Feature	Function
**Self-Guided/** **Asynchronous**	Resource Directory	A curated resource directory of available resources and services in the user’s area, e.g., food pantries, employment orgs, free medical clinics, *and substance use prevention and treatment resources.*
	Peer Created Resources	Videos, handouts, and guides to support users with navigating adulthood and building independence. Includes skill-building videos featuring the FAB to promote healthy coping and a 5-session series (Launchpad to FostrSuccess) providing practical guidance on financial literacy, housing, health and behavioral health care, college applications, and community building.
	Mood Tracking	Emoji-based daily mood tracking, which provides opportunities for self-awareness and empowers users to take control of their emotional states.
	Assessments	Assessments such as the Emotional Wellness Questionnaire (e.g., including items from the DSM-5 Level 1 Cross-Cutting Symptom measure) are available to support users in learning about their behavioral health. *National Survey on Drug Use and Health items added to provide more detailed substance use self-assessment and modified by FAB to improve accessibility.* Resources are curated based on user responses assessments.
	Missions	Support goal organization (e.g., completing specific tasks such as attending health appointments) and psychoeducation, *including a harm reduction mission to address substance use prevention.*
**Services/** **Synchronous**	Peer Support	The FAB team is available daily in-app via the “FAB Chat” to assist users with accessing resources and offer ongoing peer support and guidance.
	Navigation	Personal care navigators support users in accessing and navigating social services, healthcare, and other resources, *including substance use prevention and treatment services* to meet their basic needs.
	Clinical Care Services	Licensed clinicians provide evidence-based behavioral health services, by appointment, via telehealth, *including cognitive behavioral therapy*,* relapse prevention*,* motivational interviewing/motivational enhanced therapy to reduce substance use.*
	In-App Chat	Allows users to communicate with their care team (including the FAB).

#### Self-guided/asynchronous

The app includes both prompted and unprompted features and prompted features are typically only prompted once (unless a clinician manually prompts more times, e.g., assigns a new mission to a youth). As part of Mood Tracking (unprompted), users can log their mood daily at their own discretion by clicking on mood check-in on the main dashboard. Prompted assessments appear automatically on the user’s main dashboard based on their responses to the Resource Needs Checklist, which is completed after registration. The Resource Needs Survey asks detailed questions about resource and support needs. Additionally, the Emotional Wellness Questionnaire is shown to users who express interest in emotional wellness resources in the Resource Needs Checklist. Missions (prompted once and unprompted) such as the Harm Reduction Mission and Visual Tour Mission, are automatically assigned to all users and appear on the main dashboard alongside assessments. Users can also create their own missions, such as setting a personal goal to attend a healthcare appointment.

#### Services/synchronous

The navigation and clinical care services within the app are designed to provide users with tailored support based on their needs and preferences. A trained navigator helps users connect with essential resources such as housing, mental health care, and career planning while also assisting in skill development, including self-advocacy and time management. Users can access navigation services in multiple ways: by completing the Resource Needs Survey (RNS) or Emotional Wellness Questionnaire (EWQ)—which prompts navigators to reach out—or by initiating contact directly through the in-app chat. Navigation support ranges from quick resource referrals via chat to scheduled one-on-one Zoom sessions for goal setting and care coordination. Clinical care services are provided by licensed clinicians through telehealth appointments via. Zoom or phone. Users can access these services by requesting more information about clinical care services by messaging the FostrSpace Care team through the in-app chat, discussing the option with a navigator, or scheduling an appointment after completing the Emotional Wellness Questionnaire. During an initial session, clinicians review assessment results, conduct screenings, and develop a personalized care plan, which may include evidence-based treatments such as cognitive behavioral therapy, motivational enhancement therapy and relapse prevention. When higher levels of care are needed, clinicians coordinate referrals to external providers with the assistance of a navigator, while continuing to offer follow-up support to ensure seamless continuity of care. 

#### *ECHO*®*-FostrSpace*

The FostrSpace 101 Informational Session (30 min) includes basic youth SU psychoeducation and an introduction to the FostrSpace app (e.g., features, design, how a youth may access the app). Table 2 provides information on the six session CASAAB co-designed ECHO-FS content, which was predominantly new versus adapted content (unlike the FostrSpace app). ECHO relies on a “hub” and “spoke” model (hub represents a team of experts, learners are the “spokes”) and uses an “all teach, all learn” approach that includes peer and expert consultation and collaboration. The ECHO-FS 4-member hub team included three clinical psychologists (and study investigators) and a clinical psychology postdoctoral fellow. Each session involved a brief (2–3 min) mindfulness activity, 30-minute didactic presentation from a hub member, 10–15 min of learner questions, and a (deidentified) case presentation delivered by a different CASA learner each week (40–45 min). Case consultations focused on some aspect of SU prevention with their foster youth (40–45 min) and culminated in a tailored SU prevention plan for the CASA to use with their youth. Each session also incorporated discussion of how CASAs could refer their youth to use the FostrSpace app as additional SU prevention intervention. Topics incorporate content on primary SU prevention, harm reduction (i.e. reducing use or secondary prevention) as well as identifying when tertiary intervention (i.e., intensive substance use treatment) is needed and what modality (i.e., standard outpatient, partial or intensive outpatient program, inpatient, residential).

**Table 2:** ECHO®-FostrSpace curriculum content

Session #	Topic
1	The science behind addiction (brain development, genetics)
2	Assessment of alcohol and drug misuse (how do I identify misuse? )
3	Potential harms of substance use and harm reduction
4	Coping with trauma and youth substance use
5	Communication with your youth about substance use 1: Intro to Motivational Interviewing
6	Communication about substance use 2: Engaging Youth into Services

## Results

### Enrollment and retention

The rate of referral to study enrollment rate was 70% (10 dyadic referrals and 7 dyads enrolled). All open trial dyads (7 youth, 7 CASAs) completed the quantitative assessment that would be used in the upcoming implementation-effectiveness trial. Seven CASAs and 5 youth completed all open trial procedures, including follow-up open trial assessments focused on feasibility and acceptability. One youth withdrew from participation after completing the initial assessment due to lack of continued interest, and another youth was lost to open trial follow-up by the study team (i.e., was still involved with their CASA but not responding to study team follow-up).

### Youth characteristics

Youth were on average 14.9 years old (SD = 2.2, range = 14–19), identified their gender as young woman (*n* = 4, 57.1%) or young man (*n* = 3, 42.9%), and ethnoracially identified (check all that apply) as 71.4% (*n* = 5) Black or African American, 14.3% (*n* = 1) Hispanic or Latino, 14.3% (*n* = 1) Native Hawaiian or Other Pacific Islander, 42.9% (*n* = 3) White, and 28.6% (*n* = 2) other (Mexican/Asian/Gypsy, Middle Eastern). One youth (14.3%) identified as a Lesbian, with the remaining (71.4%, *n* = 6) identifying as Straight/Heterosexual. During the past 12 months, one youth reported they used alcohol “only a few times and two reported cannabis use (one only a few times, the other daily); youth did not report use of any other substances in the past year. On the TCU Drug Screen 5, one youth scored a 7 (suggesting the presence of a severe tobacco use disorder) and all other youth responded “no” to all symptom items, receiving a score of 0. Most youth reported they had never talked about alcohol (71.4%, (*n* = 5), marijuana (71.4%, (*n* = 5), or other drugs (85.7%, (*n* = 6) with their CASA. The few youth who had rated the discussions highly in terms of how helpful they were and how comfortable they felt (all ratings of 5–7 on scale of 1 = not at all to 7 = very).

Regarding MH, 57.1% (*n* = 4) of youth reported they had been given a MH diagnosis in their lifetime, including 42.9% (*n* = 3) attention deficit hyperactivity disorder, 14.3% (*n* = 1) bipolar disorder, 28.6% (*n* = 2) a depressive disorder, 28.6% (*n* = 2) an anxiety disorder, 14.3% (*n* = 1) psychosis, 57.1% (*n* = 4) posttraumatic stress disorder, and 14.3% (*n* = 1) other (“explosive rage disorder”). On the National Stressful Events Survey PTSD Short Scale, youth overall endorsed mild trauma symptoms (reflects an average score with a possible range of 0 to 4; actual range = 0–2; M = 0.41, SD = 0.73).

### CASA characteristics

CASAs were on average 59.8 years old (*SD* = 10.9, range = 43–72), 85.7% (*n* = 6) women and 14.3% (*n* = 1) men, and identified ethnoracially as 85.7% (*n* = 6) White and 14.3% (*n* = 1) American Indian/Alaska Native. All CASAs had a college degree, with 2 (28.6%) completing additional post-graduate education; 71.4% (*n* = 5) of CASAs were currently retired, 14.3% (*n* = 1) worked as an attorney/legal professional and 14.3% (*n* = 1) in social services. CASAs had been volunteers ranging from 6 months to 10 years and supporting the participating youth ranging from 6 months to 6 years.

All CASAs (100%) reported they had spoken to their youth about alcohol and cannabis at least once, with a majority having spoken to their youth a few (2–3) times about alcohol (71.4%; *n* = 5) and cannabis (57.1%/*n* = 4); less than half (42.9%/*n* = 3) spoke to them about other drugs and 28.6% (*n* = 2) reported they had never spoken to their youth about other drugs. CASAs tended to feel moderately comfortable talking to their youth about these substances (scale: 1 = not at all comfortable to 7 = very comfortable; alcohol: *M* = 5.9, *SD* = 1.9; cannabis: *M* = 5.7, *SD* = 1.9; other drugs: *M* = 6.4, *SD* = 0.9), though were less confident that their discussions were helpful (scale: 1 = not at all helpful to 7 = very helpful; alcohol: *M* = 3.7, *SD* = 2.1; cannabis: *M* = 3.9, *SD* = 2.0; other drugs: *M* = 5.6, *SD* = 1.1). CASAs reported low levels of confidence (scale: 1 = not at all confident to 5 = extremely confident) in their ability to use motivational interviewing strategies to get adolescents’ buy-in for SU treatment (*M* = 1.9, *SD* = 0.4, range = 1–2), talk to adolescents about SU, like potential harms and harm reduction strategies (*M* = 2.9, *SD* = 0.9, range = 2–4), determine how, when, and why to involve adolescents’ families in treatment and recovery (*M* = 2.0, *SD* = 0.6, range = 1–3), determine how, when, and why to refer adolescents to SU treatment (*M* = 1.6, *SD* = 0.5, range = 1–2), and serve as an expert in their CASA agency for questions related to SU in adolescents (*M* = 1.3, *SD* = 0.8, range = 1–3).

### Intervention engagement and feedback

#### *ECHO*®*-FostrSpace*

CASAs attended at least 75% of all sessions (attending on average 88% or 5 of the 6 active condition sessions). Session satisfaction ratings were high (83% endorsing strong satisfaction with multiple aspects of sessions). Exit interviews suggest the CASAs found the ECHO-FS content and approach feasible and acceptable as demonstrated by statements such as:

*“I loved knowing about the brain… and motivational interviewing was fabulous. I actually went and got a book on it because I just thought it was a really interesting way to communicate.”*

*“I enjoyed participating with the other CASAs and their presentations also. It was good to hear how other CASAs are mentoring and dealing with their youth. That was good.” *

“*I liked the way we had CASAs share information and you gave feedback. I think that was very helpful. Each one was individual and so even though each youth was different we were able to obtain knowledge on different situations and things that other CASA youth are going through so that in case we came across it we would be able to have similar ways to help them. So it was a lot of good feedback.*”

CASAs also noted that attending ECHO®-FostrSpace sessions significantly enhanced their ability to discuss SU with their CASA youth, fostering trust and improving communication. A majority of participants (71.4%, *n* = 5) indicated what they learned during the sessions (e.g., how SU affects the brain) enabled them to approach the topic more effectively. Another participant shared that the sessions created an opportunity to discuss their youth’s current and past SU, noting:

*“I learned new information that I probably would not have learned for a long time unless she told me, and this experience facilitated that.”*

Additionally, a participant highlighted the role of the sessions in fostering openness and trust, recounting a meaningful conversation with their CASA youth:

*“I think that came through me being able to ask questions after I went through the sessions. Just talking more openly about, you know, how she feels about what she is doing and how it might affect her goals.”*

Despite these successes, some participants encountered challenges, including fears of triggering youth’s past trauma around family of origin history with SU and initial discomfort when addressing the topic.

### FostrSpace app utilization

Of the seven youth enrolled in the study, six (86%) successfully registered and four (57%) actively engaged with the FostrSpace app (i.e., logged into the app and engaged with any features). The number of unique logins per participant ranged from 1 to 9 (modal log-in = 1). Two participants continued to use the app beyond the first day (33% of those who registered). The average youth (*n* = 4) score on the SUS over 60 days of utilization was 56 (target score of 68 or above for usable application).

### App features

Among the app features, missions, in-app chat messaging, and mood check-ins were the most frequently used, based on the total number of interactions. The mood check-in feature had the most unique users (*n* = 4), followed by missions (*n* = 3). No participants utilized direct clinical services during the open trial period. Based on elevated EWQ symptom scale scores, one participant was flagged for follow-up, prompting outreach by navigators to directly connect them with FostrSpace clinical services resources and/or external supports. Similarly, two participants were directly outreached for a navigator session based on indicated resource needs (e.g., interest in navigator support). Of these, one attended an initial session with a navigator and later reported meeting their goal, and the other engaged with in-app messaging with the navigator (to schedule a session), but was unresponsive over time and lost to follow-up. User-initiated messages accounted for 4.5% of all communication in the app, while the remaining messages were initiated by the FostrSpace Care Team, including automated welcome messages, announcements and reminders about app resources, outreach efforts, and navigation scheduling. Supplemental Table 1 provides a detailed summary of open trial app usage by individual youth participant.

### General impressions and feedback

In exit interviews, five youth (two who engaged with the app over time and three who did not use past one time) provided positive feedback about the features, design, and content of the FostrSpace app. Most participants emphasized the importance of having content developed by youth with lived foster care experience for relevance, and recognized the value of MH and SU resources even though they had not used these resources themselves. One participant noted,

“*Just having a person that knows what you are going through. That’s what foster kids need. Because foster care is going to put you through a lot of things. Kids just need to talk to someone who knows what you are going through. That’s what they really need. That knows that they are going through and isn’t just doing it for a paycheck.*”

Despite not using navigation or clinical services, all youth emphasized the value of having direct access to a live person for help, particularly in situations requiring additional support. Youth also appreciated the flexibility to either search for information independently or seek assistance as needed. For example, one youth emphasized the app’s utility for finding employment resources, sharing they used it to find an organization specializing in employment assistance and successfully connected with the organization for support. Additionally, one youth shared the app with a friend they believed could benefit from MH support. All five youth participants interviewed indicated that FAB-created content (videos, resources) covered topics that are important and relevant for foster youth. FAB resources that were available in real-time (via Zoom) to open trial participants (e.g. a synchronous workshop series called Launchpad to FostrSuccess that focused on skills development in SDoH areas, such as employment and education) were not attended. When garnering feedback on this, one participant noted,

“*Yeah, the topics seem helpful and relevant. Maybe adding information about how to manage finances would be helpful.*”

Another youth suggested offering a recorded option of the workshop could enhance accessibility. Exit interviews also provided some clarity on why they didn’t engage in navigation services:

“*I liked that you can meet someone about goals. I did one or maybe two sessions but it wasn’t for me…Because I am still in foster care and already have social workers and I didn’t need another one.*”

### Access challenges

Three youth identified significant barriers with the app log-in procedures, which hindered many from returning to use the features and content of the app. One youth shared:

“*I lost my old email account that I put the app under. So I had trouble making a new account and then the email I did want to use, it didn’t let me use for some reason so I had to use another email. And then when I logged in, literally almost two or four days later to look at the app, it logged me out. I put in the right information but it said it wasn’t the right information.*”

Another youth suggested the app’s design as a web-based platform contributed to login frustrations,

*“It would make it easier to use ‘cause everything would be in one app instead of having everything in safari. So you don’t lose your process and you don’t have to keep logging in.”*

Two youth reported not having issues with login, with one noting their experience was improved by having s0meone from the FostrSpace Team walking them through the process. External factors further limited app use among participants, including challenges like losing access to their mobile phone (*n* = 2), not consistently having access to WIFI or cellular data (*n* = 1), and securing independent time to access the app outside of required school and work activities (*n* = 2). 

### Modified login process retesting

To address feedback about existing login process, we collaborated with our technology developers to simplify login, using a magic link sign-in process. This passwordless authentication method allows users to enter their phone number on the login page and receive a unique, time-limited code via SMS-messaging, to enter into the login page and securely log the user into their account. Four youth participants tested the revised login process and reported the new feature significantly improves app access, making it much more likely they will use the FostrSpace app more frequently. Given their interest in supporting youth with FostrSpace app usage, open trial CASA participants were also asked to provide feedback on the utility of the revised log-in process; six CASAs provided feedback, unanimously indicating it would significantly improve app accessibility for youth, who often struggle to remember passwords.

## Discussion

### FostrSpace and ECHO®-FostrSpace feasibility and acceptability

Engaging in participatory co-design approaches with advisory boards comprised of the consumers of digital health interventions is key to acceptability and satisfaction with content and approach. Our open trial findings also underscore the importance of collecting and analyzing real-time user experience data to quickly identify and address barriers to app access. A recent systematic review to understand more about access and engagement in child and adolescent digital health interventions found that overall, multiple measures of engagement and access in each study are lacking but for 5 of 24 studies included, the frequency of at least one log-in ranged from 35.6 to 100% (and no studies explicitly foster youth) [50]. Our data suggest 86% of consented youth (6 of 7) registered to use the app and of those 6, 4 (67%) logged in to use the app at least once; thereby fitting within the range of existing published digital health intervention studies. Nevertheless, open trial feedback from youth participants revealed a majority encountered issues with the initial FostrSpace login process (e.g., due to password requirements). Login barriers have the potential to obfuscate trial outcomes due to their impact on youth app utilization and user experience research has documented the negative relationship between increased demand on user effort and system usability; streamlining the app login process was therefore a high priority to improve app usability and promote a positive user experience. Additional open trial data from youth and CASAs suggests addressing this technological barrier will support youth engagement and utilization in the upcoming implementation-effectiveness trial, particularly since foster youth who accessed the FostrSpace app found its resources (including missions for goal-setting, emotional wellness questionnaire for self-assessment of MH and SU prevention needs), live navigation, peer supports and access to direct clinical services appealing and relevant.

CASAs who participated in the ECHO-FS telementoring program had high attendance and also reported high levels of satisfaction with the implementation strategy content and approach; they particularly noted the direct impact on increasing their competence when communicating with youth about substances. Findings overall suggest that working within an existing system serving foster youth, such as CASA programs, has the potential to improve adult-adolescent communication about substances and to expand youth access to SU prevention resources such as FostrSpace.

### FostrSpace app: youth feedback and adaptations

Youth participants emphasized the importance of content that is tailored and created by and for foster youth, as well as the ability to access resources based on their individual needs. While all youth emphasized the importance of having access to live support through the FAB chat, navigation, and clinical services, they noted not all users require live assistance. Youth highlighted the value of offering self-guided resources while appreciating the option to receive more personalized support for more complex issues or when additional support is needed. Youth also noted the importance of distinguishing the role of care providers (e.g., navigator) from existing professionals in their lives such as social workers, who may already be working to help youth access resources. Preliminary app utilization data aligns with youth feedback, showing greater engagement with self-guided features. This is consistent with existing literature suggesting variation in usage reflects a diversity of youth needs and preferences [51]. FostrSpace can further support youth-driven exploration of SU prevention and MH care resources by maintaining a selection of self-guided options alongside available navigation and clinical services.

Youth feedback and FostrSpace app utilization data directly informed additional app adaptations in preparation for the full implementation-effectiveness trial. For example, while youth reflected on the importance of the topics covered in the synchronous Launchpad to FostrSuccess workshop series, none attended the live sessions. As such, we turned this into a series of videos users can watch asynchronously to learn about ways of addressing SDoH (e.g., financial literacy). Additionally, youth noted the importance of having people with lived experience accessible in the app, however, did not utilize the peer FAB chat. Review of our protocols revealed that beyond the initial message users receive when signing up for the app where the FAB introduces themselves, no proactive outreach by the FAB was conducted, therefore in the full trial the peer support protocol will now include weekly outreach (via in-app messaging) to users by the FAB to offer support. Youth also suggested that a native (versus web-based) app be considered for ease of access; however, other data collected in the open trial suggests there are barriers to foster youth having continued access to a mobile phone (e.g., due to resources, cellular plan disruptions and/or youth safety reasons), suggesting a web-based app platform that can be easily accessed from multiple different types of devices (e.g., laptop, desktop, ipad etc.) and settings (e.g., school, library) would promote access in times when a mobile phone may not be accessible.

### ECHO®-FostrSpace: CASA feedback and adaptations

Our participatory co-design approach with CASAs in creating the ECHO-FS curriculum also provided rich data in understanding the dearth of youth SU prevention and treatment knowledge CASAs have despite adolescent foster youth consistently reporting some of the highest rates of alcohol, cannabis and other drug use among pediatric samples and often with co-occurring (untreated) psychiatric, including traumatic stress, symptoms. Our open trial process revealed that CASAs are seeking psychoeducation on the etiology and underlying drivers of SU among foster youth as well as concrete skills related to how to speak to foster youth about SU, whether it be related to primary prevention (with youth with family history) or secondary prevention needs (for youth whom CASAs know are already using alcohol and/or drugs). Attendance and satisfaction data from CASAs participating in the 7 sessions (FostrSpace Info + ECHO-FS sessions) was high demonstrating that SU prevention is a relevant and important topic for CASAs to receive support and training on. CASA programs also require that CASAs have at least 12 h of annual relevant continuing education training, for which the ECHO-FS curriculum could count for thereby enhancing likelihood of CASA engagement and sustainability within CASA programs. The ECHO® model is a suitable implementation strategy to test in a larger trial as to whether it can lead to increased reach of the FostrSpace app. Youth SU and MH data support need for SU primary prevention services and demonstrate the relevance of the ECHO-FS content for CASAs in working with foster youth. Further, youth and CASAs diverged in their report of how much they were already talking about SU, with CASAs reporting more frequent discussions than youth. This suggests a need for building CASA volunteers’ capacity to have effective and memorable discussions related to SU with the young people they support.

### Limitations

By virtue of being an open trial design, our study sample size was very small, which does not allow us to draw conclusions from assessment measures. For example, mental health and substance use symptom prevalence and need was much less than what would be expected based on prior research with foster youth. Our FostrSpace app registration challenges may have limited what participating youth accessed in the app and therefore limited feedback about certain features within the app to only a few users; however, without the open trial we would have never learned about the registration and log-in barriers that youth were experiencing and this would have been a major impediment to testing hypotheses related to FostrSpace app uptake and utilization in the full trial. Lastly, this open trial was conducted in one CASA program (and in one county) and while it was successful and milestones were achieved, there could be new and unknown challenges when conducting the trial in new CASA programs and counties that the open trial might not have prepared us for. Continued involvement of a CASAAB and the FAB will help us to manage and problem-solve challenges as they arise. As part of the implementation science design, we will also be collecting data on organizational and system-level barriers and facilitators of intervention implementation and uptake, which will provide a rich source of guidance for the field on how to successfully leverage technology to increase access to substance use prevention interventions for foster youth.

## Conclusions

According to Whitehead and colleagues (2024) systematic review [50], few published digital health intervention studies for youth (including those focused on mental health) consider factors related to access and engagement and even fewer have tested strategies to improve access and engagement. Lessons learned from an open trial of the FostrSpace app and the ECHO-FS curriculum as an implementation strategy were critical in shaping multiple design considerations for a large-scale health services and implementation science trial of the FostrSpace app. The FostrSpace-CASA trial will be among the first to test ways in which the field might advance the science of digital behavioral health equity and prevent substance use among foster youth.

## Supplementary Information

Supplementary Material 1.

Supplementary Material 2.

Supplementary Material 3.

## Data Availability

The datasets generated and/or analysed during the current study phase are not publicly available due to the small sample size of the open trial (and therefore less protections for participants even with deidentified data), but are available from the corresponding author on reasonable request.

## Abbreviations

CASA: Court appointed special advocate
ECHO: Extension for community health outcomes
SU: Substance use
MH: Mental health
SDoH: Social determinants of health
FAB: FostrSpace advisory board
CASAAB: Court appointed special advocate advisory board
EWQ: Emotional wellness questionnaire
DSM: Diagnostic and statistical manual of mental disorders
