# Supplementary material for: Advancing access to substance use prevention for foster youth through digital innovation: an open trial of fostrspace with court appointed special advocate programs
Source: BMC Health Serv Res. 2025 May 10;25:676. doi: 10.1186/s12913-025-12811-9 (PMC12065223; doi:10.1186/s12913-025-12811-9)
Supplement: Supplementary file 1 — Supplementary Material 1. [file 12913_2025_12811_MOESM1_ESM.docx]

| **Supplemental Table 1. Individual App Utilization for Participants who Engaged with the FostrSpace app (n=4)** | | | | | | | | |
| --- | --- | --- | --- | --- | --- | --- | --- | --- |
| **Participant** |  | **Frequency of App Features Used** | | | | | **App Use Duration** | **# of Logins** |
|  |  | **Missions** | **In-App Chat** | **Mood Check-in** | **Resource Directory** | **FAB-Created Content** |  |  |
| Participant A | Used missions, mood check-in, in-app chat messaging, and navigation services. Participant completed the harm reduction, visual tour, resource needs survey, and navigator-created missions for attending navigation appointment and completing their goal. Met with the navigator once, no showed 3 times, and rescheduled 3 times. Participant stated they no longer needed navigation services, having met their goal independently. Sent 3 in-app messages to the navigator and received 24 messages from the navigator and 1 from the clinician. | 5 | 3 | 2 | 0 | 0 | 27 days | 9 |
| Participant B | Used missions, mood check-in, in-app chat messaging, resource directory. Participant completed the harm reduction, visual tour, resource needs survey and EWQ missions. One the EWQ they flagged high on 2 of the 6 symptom scales. Participant did not respond to navigator or clinician outreach attempts to follow-up on elevated symptom scales. Expressed interest in resources but left navigation support question blank. Navigator scheduled appointments to discuss navigation support and clinician connection due to EWQ flags. Participant no-showed once and rescheduled twice but never attended. Sent 3 in-app messages to the navigator and received 17 navigator messages and 2 clinician messages. | 4 | 3 | 3 | 1 | 0 | 40 days | 5 |
| Participant C | Used missions and mood check-ins but did not log in again after the initial session. | 2 | 0 | 1 | 0 | 0 | 1 day | 1 |
| Participant D | Used mood check-in once but did not log in again after initial session. | 0 | 0 | 1 | 0 | 0 | 1 day | 1 |
