# Supplementary material for: Advancing access to substance use prevention for foster youth through digital innovation: an open trial of fostrspace with court appointed special advocate programs
Source: BMC Health Serv Res. 2025 May 10;25:676. doi: 10.1186/s12913-025-12811-9 (PMC12065223; doi:10.1186/s12913-025-12811-9)
Supplement: Supplementary file 2 — Supplementary Material 2. [file 12913_2025_12811_MOESM2_ESM.docx]

R61 Aim 3 Qualitative Individual Interview Guide with Youth Participants (3 months) to refine Cluster Randomized Controlled (RCT) design

Thank you so much for agreeing to meet with us to share your thoughts and experiences about the FostrSpace app. You have been involved in this study for [insert time, i.e., about 3 months] and we’d really like to hear more from you about what has gone well and what you think could improve. For example, it will be really helpful to understand any specific feedback you have on how FostrSpace could be tailored to meet your specific needs.

You are the expert in this area and I am hoping that you will share your experiences with me. I am interested in your opinions and there are no right or wrong answers.

Some of the questions might ask you to share your personal experiences and I really appreciate your honesty in answering them. However, please do not feel like you have to respond to every question. If you want to follow-up on something or give an example, please feel free to do so. I will be recording this session so that I don’t miss any of your comments. No names will be used when reporting on the findings from this study.

Instead, we will replace your name (and names of anyone mentioned) with a false name, or report themes from responses in aggregate. Your responses are completely confidential. If you have a cell phone, please put it on silent and if you absolutely need to answer please just let me know and we can pause the interview. This interview should take about 30 minutes to complete. Any questions before we get started?

[*Start the recorder and state the* ***participant ID****, the* ***date of the interview****, and the*

***name of the facilitator/interviewer****.*]

Let’s start by talking about the FostrSpace app. [[link](https://ucsf.box.com/s/avgdwk32oh4upb3v64e6fzea1g4qo829) to the app screenshots]

1. In general, what did you find the most helpful about the FostrSpace app?
2. What did you find not so helpful about FostrSpace?
3. What did you think of the app itself in terms of features, usability, youth friendliness?
4. If you could change or tailor any aspect of the app, what would it be, and why?
5. Describe how your CASA worker first introduced you to the app
6. How did your CASA worker help to set you up to use the FostrSpace app? [prompts: query about method, timing of referral, tangible supports]
7. How did you use the FostrSpace app over the past few months? Describe how you might have used specific features within the app (e.g., passive resources, navigator, FAB chat, adulting 101, clinical intervention services/therapy).
   1. What aspects of the app were you most drawn to and why?
8. How did your CASA worker use FostrSpace to support you with your emotional wellness and/or substance use prevention goals? [If responds they did not help, then how might your CASA worker use FostrSpace to support you in these areas?]
9. In what ways did FostrSpace support you in accessing substance use/mental health/emotional wellness services, if any?
10. Part of what we’re interested in is to see how CASAs might be able to help youth like yourself use apps to support their goals; How did you and your CASA continue to use FostrSpace together over the course of the past few months?
11. "Sometimes, who we are can affect how comfortable we feel using certain apps. How was that for you with FostrSpace? Did you feel like any parts of your identity were really respected in the app? Also, what do you think about using apps for mental health, emotional wellness, and preventing substance use? How does that connect to your experience?"
12. "How did your identity impact your experience with FostrSpace? Did you feel respected in any way? What do you think about using apps for mental health and substance use prevention?"
13. It’s also the case that things outside our control can impact how we use technology. Can you talk about anything in your life that impacts your ability to use FostrSpace? [prompts: stigma, social determinants of health/SDOH, hospitalization/detention, loss of phone plan]
14. What about CASA as a program influenced how you used FostrSpace? [prompts: integrated into CASA workflow and orientation, CASA workers brought it up in first interaction, CASA worker never introduced it]
15. Did you attend any of the Launchpad to Success workshop sessions? Did you receive (or see) and of the workshop invites? What would make it easier for you to attend these workshops? [link of [workshop topics](https://ucsf.box.com/s/l670dkl0vlr0njjh2vp6lhyxfxloq0ok) for facilitator only]

Question about baseline and follow up survey completion:

1. Was there anything that would have helped you with completing the research surveys that you were asked to complete when you first joined the study and at follow up (30 and 60 days)?

Is there **anything I might not have asked** you about the FostrSpace app that you’d like to share?

Thank you so much for your time today. We look forward to using your feedback to make the FostrSpace app more suitable to addressing substance use and mental health needs of young people.
