# Supplementary material for: Advancing access to substance use prevention for foster youth through digital innovation: an open trial of fostrspace with court appointed special advocate programs
Source: BMC Health Serv Res. 2025 May 10;25:676. doi: 10.1186/s12913-025-12811-9 (PMC12065223; doi:10.1186/s12913-025-12811-9)
Supplement: Supplementary file 3 — Supplementary Material 3. [file 12913_2025_12811_MOESM3_ESM.docx]

R61 Aim 3 Qualitative Individual Interview Guide with CASAs to refine Cluster Randomized Controlled (RCT) trial design

Thank you so much for agreeing to meet with us to share your thoughts and experiences in ECHO-FostrSpace and supporting your youth in using the FostrSpace app. You have been involved in this study for [about 3 months] and we’d really like to hear more from you about what has gone well and what you think could improve. For example, it will be really helpful to understand any specific feedback you have on ECHO content as well as the telementoring approach.

You are the expert in this area and I am hoping that you will share your experiences with me. I am interested in your opinions and there are no right or wrong answers.

Some of the questions might ask you to share your personal experiences and I really appreciate your honesty in answering them. However, please do not feel like you have to respond to every question. If you want to follow-up on something or give an example, please feel free to do so. I will be recording this session so that I don’t miss any of your comments. No names will be used when reporting on the findings from this study.

Instead, we will replace your name (and names of anyone mentioned) with a false name, or report themes from responses in aggregate. Your responses are completely confidential. If you have a cell phone, please put it on silent and if you absolutely need to answer please just let me know and we can pause the interview. This interview should take about 30 minutes to complete. **The UCSF research team will follow up with you in the following weeks to find an additional 10-15minutes brief interview to get additional feedback from you about the FostrSpace app. Our team recently received very helpful feedback from the youth participants, and we are working with our developer to implement some of these changes and would like to share with you those updates and get your feedback.** Any questions before we get started?

[*Start the recorder and state the* ***participant ID****, the* ***date of the interview****, and the*

***name of the facilitator/interviewer****.*]

First, I will ask you a question about the FostrSpace app:

1. Could you tell me how to access the FostrSpace app? [explain how you would access the app in your phone/device]

Now, I will ask you general questions about ECHO-FostrSpace and we will wrap up with questions specifically about the FostrSpace app.

1. What was your **overall impression** of ECHO-FostrSpace? [What would they like to see changed/added]
   1. Screenshare ECHO-Fostrspace [[[session table](https://ucsf.box.com/s/eawh5qk00x6br9f5stj2bkadci4efqc5)](https://ucsf.box.com/s/eawh5qk00x6br9f5stj2bkadci4efqc5)](https://ucsf.box.com/s/eawh5qk00x6br9f5stj2bkadci4efqc5):
      1. Which of the six sessions did you find useful?
      2. Which of the six sessions did you not find useful?
   2. What do you think about having videos included in the sessions?

[helpful vs not helpful]

1. What was your experience with the **telementoring approach** used throughout ECHO-FostrSpace? [prompts: helpful vs. not so helpful, peer versus hub facilitated learning]
2. What did you **learn the most** by engaging in the ECHO?
3. What were your experiences **talking about substance use** with your CASA youth? [prompts: things that made it harder or easier]
4. Do you have general feedback about **the overall didactic content** presented in ECHO-FostrSpace? [helpful vs. not so helpful]
5. Do you have general feedback about **the consultation model**? [helpful vs. not so helpful]
6. Do you have general feedback about **the case form**? [helpful vs. not so helpful]
7. Do you have general feedback about **the substance use recommendation form**? [helpful vs. not so helpful]

Thanks for your feedback on the ECHO-FostrSpace program, I want to ask you just a few more questions about the FostrSpace app specifically.

1. Have you **referred any youth** to use the FostrSpace app over the past few months?
   1. What is your sense on how the FostrSpace app helped?
   2. What barriers to using the app came up?
      1. What do you think may have impacted a **youth’s decision to use** FostrSpace? [prompts: ease of use, youth friendly, design]
   3. What made it easier to use the app?
2. Which **specific youth** do you think FostrSpace is most relevant for? Helpful to?
3. What are some **factors outside your control** influencing FostrSpace use? [prompts: stigma about substance use, hospitalization, loss of phone plan]
4. What aspects of the **CASA program** influence how FostrSpace was used to address substance use needs? [prompts: referral policies, CEU training, CASA infrastructure for technology]

Is there **anything I might not have asked** you about ECHO-FostrSpace or the FostrSpace app that you’d like to share?

Thank you so much for your time today. We look forward to using your feedback to make ECHO-FostrSpace and the FostrSpace app more suitable to addressing substance use and mental health needs of young people.
